# FACTORS ASSOCIATED WITH COGNITIVE FUNCTION AMONG OLDER ADULTS IN VIETNAM

**DOI:** 10.1093/geroni/igac059.2067

**Published:** 2022-12-20

**Authors:** Yasuhiko Saito, Choy Lye Chei, Nguyen Vu, Linh Dong

**Affiliations:** Nihon University, Chiyoda-ku, Tokyo, Japan; Nihon University, Chiyoda-ku, Tokyo, Japan; Institute of Population, Health and Development, Hanoi, Ha Noi, Vietnam; Institute of Population, Health and Development, Ha Noi, Ha Noi, Vietnam

## Abstract

While Viet Nam is aging rapidly, there are few studies on cognitive function of older adults. We aimed to study status of cognitive function among older adults in Viet Nam and examine factors associated with cognitive function. The baseline data of the Longitudinal study of Ageing and Health in Viet Nam conducted in 2018/2019 was used for the study. LSAHV was a nationally representative survey of those aged 60 and above and computer-assisted personal interview (CAPI) using a tablet was conducted for data collection. The number of older person included in the study was 5,530. Cognitive function was measured by Short Portable Mental Status Questionnaire (SPMSQ). Factors such as demographic, socioeconomic, health behavior and daily activities such as reading newspaper were examined for their association with cognitive function by linear regression analyses. We found that better cognitive function was associated with engaging in physical activity in both male and female, listening to radio and receiving material support from children in male, and reading in female. Poorer cognitive function was associated with IADL limitation and financial support in both male and female, obesity in male, and hypertension and hearing loss in female. Our study provides an understanding of the associated factors with cognitive function of older people in Viet Nam. Some of the factors examined are modifiable and can be improved to prevent cognitive decline. It is also important to consider sex differences in planning and implementation of interventions and programs to prevent cognitive decline in Viet Nam.

